# Characterisation of HIV‐1 Gag Cytotoxic T‐Lymphocyte Epitopes in the Southern African Region—A Systematic Review

**DOI:** 10.1111/imm.70156

**Published:** 2026-05-31

**Authors:** Tumelo L. Fortuin, Paballo Nkone, Shayne Loubser, Caroline T. Tiemessen, Simnikiwe H. Mayaphi

**Affiliations:** ^1^ Department of Medical Virology University of Pretoria Pretoria South Africa; ^2^ Waterlab (Pty) Ltd Pretoria South Africa; ^3^ National Institute for Communicable Diseases and Faculty of Health Sciences University of the Witwatersrand Johannesburg South Africa; ^4^ National Health Laboratory Service‐Tshwane Academic Division (NHLS‐TAD) Tshwane South Africa

**Keywords:** conserved immunodominant epitopes, HIV‐1 gag CTL epitopes, HIV‐1 subtype C, southern African region

## Abstract

During early HIV‐1 infection, robust Cytotoxic T‐lymphocyte (CTL) responses are mostly targeted at immunodominant Gag p24 epitopes to reduce HIV‐1 viraemia to a set‐point. The aim of this study was to review the current body of knowledge on HIV‐1 Gag CTL epitopes in the southern African region where subtype C is prevalent. Peer‐reviewed records were obtained from three databases: PubMed Central, Web of Science Core Collection, and Scopus, using the following search terms: HIV subtype C Gag epitopes, and HIV clade C Gag epitopes. The search results were restricted to countries within the southern African region, and only data published in English and between the years 2000–2025 were considered for this review. The search from the three databases produced a total of 2103 peer‐reviewed records, and 49 records were included in the review. The majority of studies (58.44%) were conducted in South Africa, followed by Botswana (15.58%), Zambia (10.39%), Malawi (7.79%), Zimbabwe (6.49%) and Angola (1.30%). There were no studies identified from other southern African countries. A total of 60 Gag CTL epitopes were identified, of which 17 (28.33%) were located within the matrix protein (p17), 33 (55.00%) within the capsid protein (p24), and 4 (6.67%) within the Gag polyprotein (p2p7p1p6). The commonly detected immunodominant epitopes were mostly located within the Gag p24 protein; and included TPQDLNTML (TL9, Gag p24 48–56) and TSTLQEQIGW (TW10, Gag p24 108–117) present at 16.00% and 13.3%, respectively. The proportion of HLA‐A, B and C allotypes in this systematic review were 18%, 78%, and 4%, respectively. The more common HLA‐B allotypes that restrict immunodominant Gag epitopes and facilitate better control of HIV‐1 were HLA‐B*57, ‐B*58:01, ‐B*42:01 and ‐B*81:01. This systematic review has provided important insights into the description of immunodominant Gag epitopes and HLA‐I alleles that contribute to the control of HIV‐1 viraemia in the southern African region. It has also exposed that some CTL epitopes identified in the southern African studies are not reported on the Los Alamos HIV database (LANL HIV database). This highlights a need to have this database updated with this information as it is used as a reference for epitopes. This review could provide insights into the design of an epitope‐based HIV‐1 vaccine that would also be effective in the southern African region.

## Graphical abstract

Distribution of studies on HIV‐1 Gag cytotoxic T‐lymphocyte epitopes in the southern African region showing that the majority of studies (58.44%) were conducted in South Africa, followed by Botswana (15.58%), Zambia (10.39%), Malawi (7.79%), Zimbabwe (6.49%), and Angola (1.30%). There were no studies found in Eswatini, Lesotho, Madagascar, Mozambique, and Namibia.

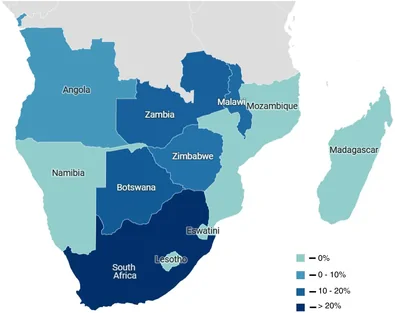

**Abbreviations**
%: percent
°C: degree celsius
Aa: amino acid
AIDS: Acquired Immunodeficiency Syndrome
ART: antiretroviral therapy
ARV: antiretroviral
CD4: cluster of differentiation 4
cDNA: complementary deoxyribonucleic acid
CTL: cytotoxic T lymphocytes
DNA: deoxyribonucleic acid
*Gag*
: group‐specific antigen
HIV‐1: human immunodeficiency virus type‐1
HLA: human leukocyte antigen
ICS: intracellular cytokine staining
IFN‐γ ELISpot: Interferon‐gamma Enzyme Linked Immuno‐Spot
IgG: immunoglobulin G
Kb: kilobase
LANL: Los Alamos National Laboratory
Ltd.: limited
LTR: long terminal repeats
MA (p17): matrix protein
NC: nucleocapsid protein
ND: not documented
NRF: National Research Foundation
ORF: open reading frame
p24: capsid protein
p2p7p1p6: gag polyprotein
PLWH: People living with Human Immunodeficiency virus
PRF: Poliomyelitis Research Foundation
PRISMA: Preferred Reporting Items for Systematic reviews and Meta‐Analyses
PROSPERO: International Prospective Register of Systematic Reviews
Pt: participant
RIS: Research Information Systems
RNA: ribonucleic acid
RT: reverse transcriptase
UNAIDS: The Joint United Nations Programme on HIV/AIDS
VL: viral load
WHO: World Health Organisation
ZA: South Africa
μl: microliter

## 1 Introduction

The HIV‐1 pandemic remains one of the global health challenges causing significant morbidity and mortality. UNAIDS estimates that there were 40.8 million people living with HIV (PLWH), 1.3 million new HIV‐1 infections, and 630 000 AIDS‐related deaths globally, by the end of 2024 [1, 2, 3]. The southern African region has been disproportionately affected and carries the highest burden of HIV‐1 infections, with South Africa being the most affected with 7.8 million PLWHIV by the end of 2022 [4, 5]. Although antiretroviral therapy (ART) treatment has improved clinical management of HIV‐1, treatment does not lead to a cure, and its long‐term effectiveness is still threatened by factors such as social stigma, adherence, as well as the emergence of drug resistance. These further emphasise the importance to develop a safe and efficacious vaccine to eradicate HIV‐1 [6, 7, 8, 9, 10].

Cytotoxic T‐lymphocyte (CTL) responses targeting conserved immunodominant Gag epitopes play an important role in the reduction of plasma viraemia to set‐point during early HIV‐1 infection [11, 12]. Epitopes such as ISW9, KF11, TL9, and TW10, located within p24, have been linked with better control of HIV‐1; moreover, these epitopes have been previously described in individuals who are able to spontaneously control HIV‐1 in the absence of therapy. Protective Human Leukocyte Antigen class I (HLA‐I) allotypes from the A03, B07, and B58 supertypes are known to restrict these epitopes in acute HIV‐1 infection, suggesting that there may be a hierarchy in which immunodominant epitopes are preferentially targeted during early HIV‐1 infection, while subdominant epitopes are targeted at later stages of infection [13, 14, 15, 16, 17, 18, 19].

HLA‐I allotypes mediate immune responses through presentation of viral peptide epitopes to cytotoxic T‐lymphocytes (CTL), however, HIV‐1 is able to evade CTL responses through the selection of immune escape variants [20, 21]. HLA supertypes are groups of HLA proteins with similar peptide binding motifs and therefore bind identical or similar peptide epitope sequences [22]. Some HLA‐I allotypes such as HLA‐A*74:01, ‐B*39:10, and ‐B*81:01, ‐B*57, and ‐B*58:01 allotypes are known to be associated with favourable disease outcomes in HIV‐1 subtype C infection [16, 23, 24, 25, 26]. Conversely, HLA‐A*36:01, ‐B*42:02, ‐B*45:01 and ‐B*58:02 allotypes have been shown to be disease susceptible in individuals with HIV‐1 subtype C infection from the southern African region [16, 22, 27, 28]. The aim of this study was to review the current body of knowledge on HIV‐1 Gag CTL epitopes in the southern African region where subtype C is prevalent.

## 2 Methods

This systematic review was performed in alignment with the Preferred Reporting Items for Systematic reviews and Meta‐Analyses (PRISMA) 2020 guidelines; however, the analysis has not been registered with the International Prospective Register of Systematic Reviews (PROSPERO). Peer‐reviewed records were obtained from three databases: PubMed Central, Web of Science Core Collection, and Scopus, using the search terms: HIV Gag subtype C epitopes, and HIV Gag clade C epitopes. The search results were restricted to countries within the southern African region (Angola, Botswana, Lesotho, Madagascar, Malawi, Mozambique, Namibia, South Africa, Eswatini, Zambia, and Zimbabwe), mainly due to the higher degree of similarity of *HLA‐I* alleles in these populations, and the predominance of HIV‐1 subtype C [16, 29, 30]. Only data published in English and between the years 2000 to 2025 were considered for this review.

Briefly, after a search was performed on a database, the search results were downloaded in Research Information Systems (RIS) format and imported into the Covidence online software tool (https://app.covidence.org), wherein three independent reviewers performed the screening and reviewing process. Initially, Covidence screened and removed duplicates using the automated deduplication algorithm. Thereafter, titles and abstracts were screened, and irrelevant records were excluded. Full‐text articles were then reviewed and studies which did not meet the inclusion criteria were excluded. For inclusion, studies had to have reported on Gag CTL epitopes and HIV‐1 subtype C in the southern African region. HLA‐I typing and functional assay data were also assessed, although studies without these data were not excluded. Vaccine trial studies were also excluded in the final analysis as this review focussed on Gag CTL responses stimulated through a natural HIV‐1 infection. Further analysis involved assessing the study participants race and sex in those studies which reported it, and the Gag epitopes detected from natural CTL responses. The Los Alamos HIV database (LANL HIV database) was used to confirm the HIV‐1 subtype C Gag epitope names, sub‐proteins and HXB2 positioning, and sequences. The restricting HLA‐I allotypes were reported directly from the respective studies. HLA‐C allotypes were reported according to the standardised World Health Organisation (WHO) Nomenclature Committee for Factors of the HLA System, as such ‘HLA‐Cw’ used in older serological studies has been reported as ‘HLA‐C' [31]. LANL HIV PepMap Tool was used to obtain the HXB2 aa numbering for Gag peptide epitopes which were not reported in the respective articles or not listed on LANL HIV database. LANL HIV PepMap Tool estimates the HXB2 positioning of peptides by comparing the similarity between the query peptide and reference peptide sequence [32].

## 3 Results

### 3.1 Search Summary

**FIGURE 1:**
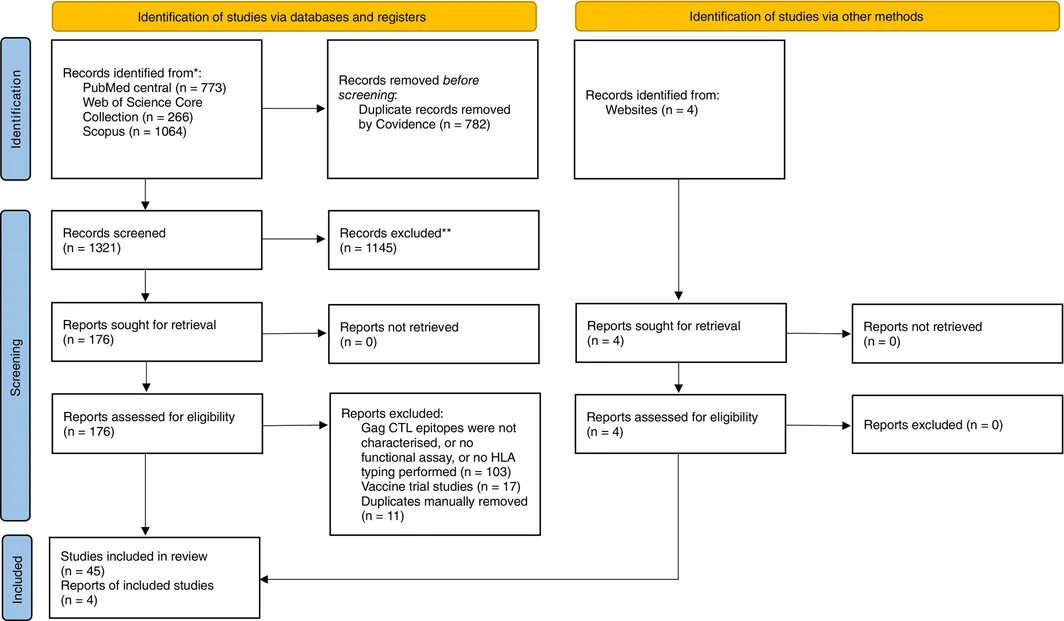
PRISMA algorithm showing the screening and enrolment of studies into this systematic review.

The search from the three databases produced a total of 2103 peer‐reviewed records which included full‐text articles and abstracts published in English between the years 2000 and 2025. Initially the records were uploaded onto Covidence which excluded 782 duplicates. Thereafter, the titles and abstracts of 1321 studies were screened, and 1145 records were excluded. 176 records were then evaluated for eligibility, and 131 studies were excluded as they did not meet the inclusion criteria. Four records were obtained through a separate search, and after evaluation were subsequently included. The screening process resulted in a total of 49 studies being included in the systematic review (Figure 1).

### 3.2 Distribution of Studies by Geographic Location Within the Southern African Region

**FIGURE 2:**
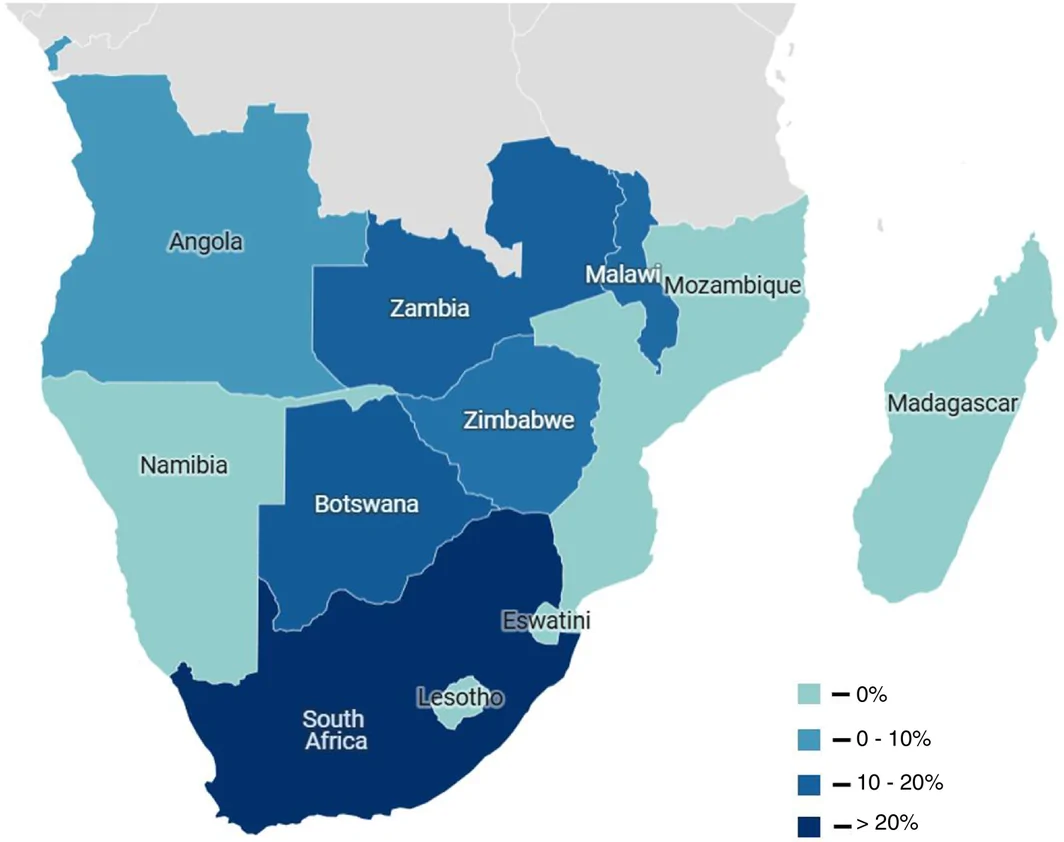
Distribution of studies within the southern African region following review showing that the majority of studies (58.44%) were conducted in South Africa, followed by Botswana (15.58%), Zambia (10.39%), Malawi (7.79%), Zimbabwe (6.49%), and Angola (1.30%). There were no studies found in Eswatini, Lesotho, Madagascar, Mozambique, and Namibia.

From the 49 studies included in the systematic review, the majority of studies (58.44%) were conducted in South Africa. There were no studies identified from Eswatini, Lesotho, Madagascar, Mozambique, and Namibia (Table 1, Figure 2).

**TABLE 1:** Studies which characterised naturally stimulated HIV‐1 Gag CTL epitopes in the Southern African region between the years 2000 and 2025.

*n*	Race	Sex	Southern African region	HLA‐I typing	Functional assay	Gag epitopes characterised	HIV subtypes	Referenced on LANL HIV database	References
81	Black, White, Haitian, Hispanic	ND	South Africa*	Yes	IFN‐γ ELISpot, tetramer staining, Chromium release assay	RK9, SL9, RY11, TL9	B, C	No	[33]
74	ND	ND	Botswana	Yes	IFN‐γ ELISpot	TL9, RY11	C	Yes	[34]
105	ND	ND	Botswana	Yes	IFN‐γ ELISpot	TL9, YL9	C	No	[35]
706	Black	Females only	South Africa, Zimbabwe, Angola, Malawi, Zambia, and Botswana	Yes	IFN‐γ ELISpot	YL9	C	No	[28]
311	Black	Females only	South Africa*	Yes	IFN‐γ ELISpot	TW10	B, C	Yes	[36]
46	ND	ND	Malawi, Zambia, Zimbabwe, and South Africa	No	IFN‐γ ELISpot	KK9, RK9, KW9, WF9, RY11, KF11, YL9, SL9	C	No	[19]
38	ND	ND	Malawi, Zimbabwe, Zambia, and South Africa	Yes	IFN‐γ ELISpot, ICS, Chromium release assay	HL9, LF11, RY11, LY9, TL9, DA9, WF9, YL9, PF10, TD10	C	No	[37]
117	ND	ND	South Africa*	Yes	IFN‐γ ELISpot, ICS, Chromium release assay	KF11, ISW9	B, C	Yes	[38]
417	ND	ND	South Africa*	Yes	IFN‐γ ELISpot	VF9	B, C	Yes	[39]
520	Black	ND	South Africa, and Zambia	Yes	IFN‐γ ELISpot	ISW9, TW10, KF11	C	No	[40]
649	ND	ND	South Africa*	Yes	IFN‐γ ELISpot	TW10, TL9, KF11, LF11, HA9, GI11, LY9, QW9, NI9, NL11, RY11, EL9, VF9, DI8, SL9	B, C	Yes	[41]
578	Black	Females only	South Africa	No	IFN‐γ ELISpot	GI11, RI9	B, C	No	[42]
578	Black	Females only	South Africa	Yes	IFN‐γ ELISpot	KF11, TW10	C	No	[43]
5	ND	ND	South Africa	No	ICS	RM9, IL10, RY11, SL9, TL9	C	No	[19]
608	ND	ND	South Africa*	Yes	IFN‐γ ELISpot, Chromium release assay, tetramer staining	KF11	B, C	Yes	[44]
21	Black, White, Indian	Female only	South Africa	Yes	IFN‐γ ELISpot	TW10, ISW9, KF11, RY11, TL9, VF9, YL9, EL9	C	No	[45]
710	ND	ND	South Africa	Yes	IFN‐γ ELISpot	ISW9, KF11, TW10, TL9, NY10, DA9, QW9, YL9, GL9	C	No	[46]
261	Black	Females only	South Africa	Yes	IFN‐γ ELISpot	TL9, TW10	C	Yes	[47]
823	Black	ND	South Africa and Zambia	Yes	None	ISW9, TW10, KF11	C	No	[48]
117	ND	ND	Zambia*	Yes	IFN‐γ ELISpot, ICS	FF8, NL9, CK9, SY9	B, C	No	[49]
778	ND	ND	South Africa	Yes	IFN‐γ ELISpot	YL9	C	Yes	[50]
9	Black	7 Females, 2 Males	South Africa	Yes	IFN‐γ ELISpot	TW10, ISW9, QW9, KF11	C	No	[51]
53	Black, White, Indian	Females only	South Africa	Yes	IFN‐γ ELISpot	AS9, TL9, EL9, TW10, YL9	C	No	[52]
63	ND	ND	South Africa	No	IFN‐γ ELISpot	ISW9, KF11, QW9, TW10	C	No	[53]
406	ND	ND	South Africa	Yes	None	TL9, QW9	C	No	[54]
36	Black, White, Indian	Females only	South Africa	Yes	IFN‐γ ELISpot	TW10, KF11, RY10, SL9, DL9	C	Yes	[55]
749	ND	ND	South Africa, and Zambia	Yes	None	TW10, TL9, ISW9	C	No	[56]
2126	ND	ND	South Africa, Botswana, Malawi and Zimbabwe*	Yes	IFN‐γ ELISpot, tetramer staining	GR11, QR10, KR9	C	No	[57]
22	ND	11 Females, 11 Males	South Africa*	Yes	IFN‐γ ELISpot, tetramer staining, Chromium release assay	KK10, KF11, ISW9, SL9, TL9, DI8, LY9, VF9	B, C	No	[24]
20	ND	58% Females	South Africa	Yes	IFN‐γ ELISpot	RY11, VF9, RY10, RL11	C	Yes	[58]
60	ND	65% Females	South Africa and Botswana	Yes	IFN‐γ ELISpot	AW11, TL9, QW9, RY11, TW10, KF11, DI8, EL8, VF9, AF8, LY9, YL9, VL10	C	No	[15]
2126	ND	ND	South Africa, Botswana, Malawi and Zimbabwe*	Yes	LANL, IFN‐γ ELISpot	KK9, KW9, RY11, LY9, TK8, IL10, VL10, ISW9, KF11, EL9, TL9, TW10, NY10, QW9	C	Yes	[22]
2093	ND	ND	South Africa and Botswana	Yes	IFN‐γ ELISpot, tetramer staining	RM9, TL9, GL9, GF9	C	No	[14]
2093	ND	ND	South Africa and Botswana*	Yes	IFN‐γ ELISpot, tetramer staining	ISW9, KF11, TW10, QW9, LF11, DW10	C	Yes	[13]
3132	ND	ND	South Africa and Botswana*	Yes	IFN‐γ ELISpot, ICS, tetramer staining	HA9, NY10, PY9	B, C, and mixed subtypes	Yes	[59]
62	Black, White, Indian	Females only	South Africa	Yes	IFN‐γ ELISpot	TL9	C	Yes	[24]
5	Black, White, Indian	Females only	South Africa	Yes	LANL	TW10	C	No	[60]
2836	ND	ND	South Africa and Botswana*	Yes	IFN‐γ ELISpot, ICS, tetramer staining	TA10	B, C	Yes	[61]
17	Black, White, African American	7 Females, 10 Males	South Africa and Malawi*	Yes	IFN‐γ ELISpot, Predicted using LANL	TL9, ISW9, YL9, TW10	B, C	Yes	[62]
2718	ND	ND	South Africa*	Yes	IFN‐γ ELISpot, tetramer staining	GL9, SV9, RM9, TL9, HA9	B, C, and mixed subtypes	Yes	[63]
3298	ND	ND	Botswana*	Yes	IFN‐γ ELISpot	KF11	B, C	Yes	[64]
22	ND	58% Females	South Africa	No	LANL	HA9, DW10, ISW9, RY11, LY9, SL9	C	No	[65]
1327	ND	ND	South Africa and Botswana*	No	IFN‐γ ELISpot	TL9	C	No	[66]
18	Black	60% Females	South Africa	Yes	IFN‐γ ELISpot	TW10, TL9, EV9, VF9, YL9, HW9, RY11, AW11, RY10, SL9	C	No	[67]
70	Black	Females only	South Africa	Yes	IFN‐γ ELISpot	TW10, QW9, NL11, EL8, ISW9, WF9, EI8, KF11, GL9, TL9, DI8, SL9	C	Yes	[68]
11	ND	7 Females, 4 Males	South Africa	Yes	IFN‐γ ELISpot	SK10, TL9, YL9	C	Yes	[69]
49	ND	ND	South Africa*	Yes	Tetramer staining, IFN‐γ ELISpot	DA9	B, C	No	[70]
21	ND	Females only	South Africa	Yes	IFN‐γ ELISpot, ICS, tetramer staining	TL9	C	No	[71]
13	ND	ND	Zambia and South Africa*	No	IFN‐γ ELISpot	SL9	A, C, D	No	[72]

*Note:* * ‐ Other participants enrolled in this study were from countries located outside the Southern African region.ICS—Intracellular cytokine staining; IFN‐γ ELISpot—Interferon gamma enzyme linked immunospot assay; LANL HIV database– Los Alamos Human Immunodeficiency Virus immunology database; et al. – and others.

Abbreviations: CTL, Cytotoxic T‐lymphocyte; et al., and others; Gag, Group‐specific antigen; HIV‐1, Human Immunodeficiency Virus type 1; HLA‐I, Human Leukocyte Antigen class I; *n*, number of participants from study; ND, not documented.

### 3.3 HIV‐1 Gag CTL Epitopes Reported in the Southern African Region

**FIGURE 3:**
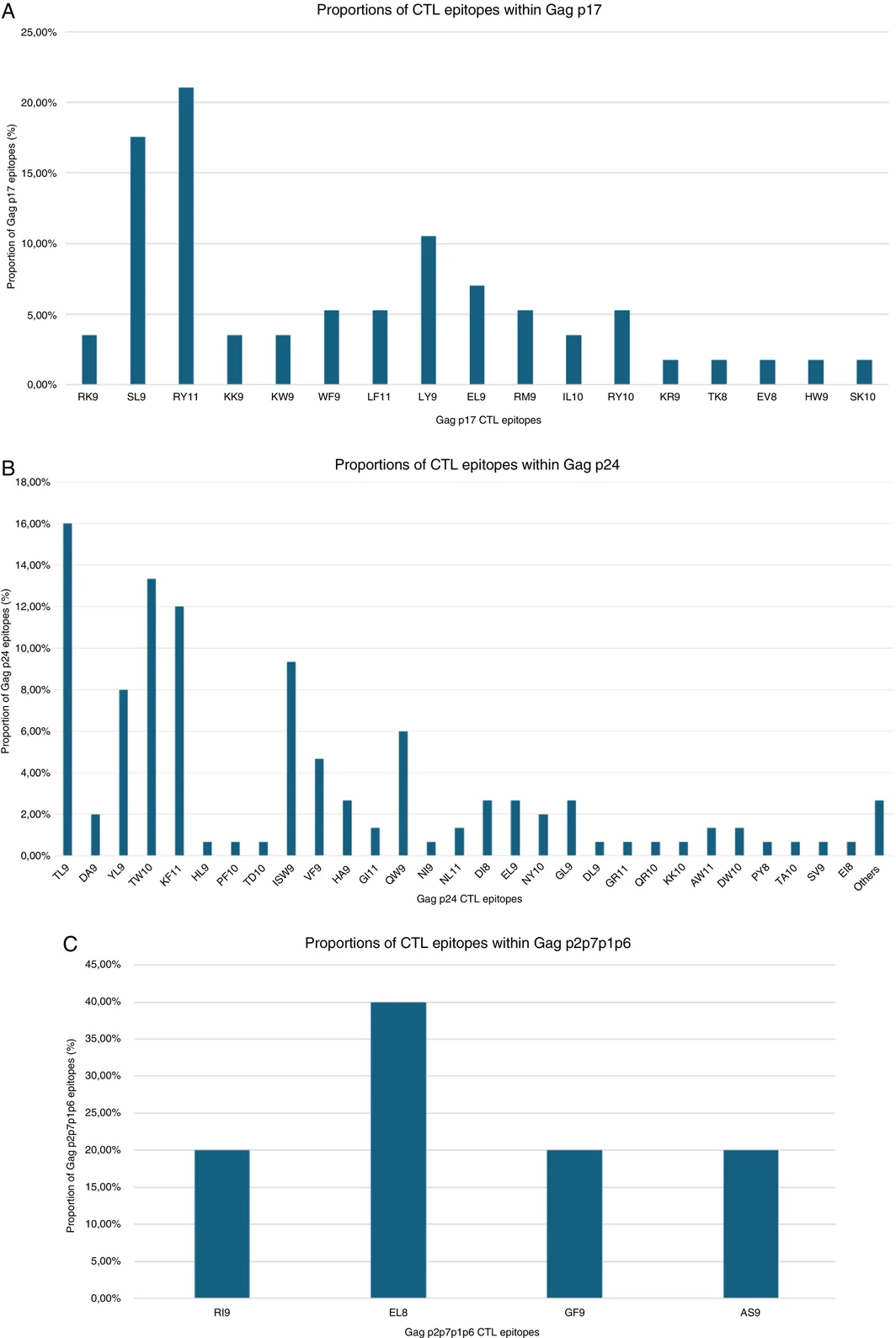
Proportions of identified Gag CTL epitopes from studies in the southern African region; (A) within Gag p17 protein, (B) within Gag p24 protein, and (C) within the Gag p2p7p1p6 protein complex. CTL, Cytotoxic T‐lymphocyte; Gag, Group‐specific antigen; p17, Matrix protein; p24, Capsid protein; p2p7p1p6, Gag polyprotein.

From the 49 studies, a total of 60 Gag CTL epitopes were identified in various proportions. From the observations made, 17 (28.33%) epitopes were located within the matrix protein (p17), 33 (55.00%) were located within the capsid protein (p24), and 4 (6.67%) fell within the Gag polyprotein (p2p7p1p6). There were 6 (10.00%) Gag epitopes which were not categorised into any of the three sub‐proteins. Within the matrix protein (p17), RSLYNTVATLY (RY11, Gag p17 76–86) and SLYNTIATL (SL9, Gag p17 77–85) were the most commonly detected Gag p17 epitopes at 21.05% and 17.54%, respectively. This was followed by LFNTVATLY (LY9, Gag p17 78–86) at 10.53%, and EPKDREPL (EL9, Gag p17 93–101) at 7.02%. The remaining epitopes were less frequently detected and appeared at proportions of approximately 5% or less (Table 2, Figure 3).

**TABLE 2:** HIV‐1 Gag CTL epitopes from studies included in the systematic review, and the HLA‐I allotypes which were documented to restrict these epitopes by the particular study.

Gag epitopes	Gag position	Gag epitope sequence	Restricting HLA‐I allotypes	References
RK9	p17 (19–29)	RLRPGGKKK	A*03	[33]
SL9	p17 (77–85)	SLYNTVATL	A*02	
RY11	p17 (76–86)	RSLYNTVATLY	A*30	
TL9	p24 (48–56)	TPQDLNTML	B*42:01	
TL9	p24 (48–56)	TPQDLNTML	B*42:01, B*81:01	[34]
RY11	p17 (76–86)	RSLYNTVATLY	A*30:02	
YL9	p24 (164–172)	YVDRFFKTL	B*15:10	
TL9	p24 (48–56)	TPQDLNTML	B*42:01, B*81:01	[35]
YL9	p24 (164–172)	YVDRFFKRL	B*15:10	
YL9	p24 (164–172)	YVDRFFKRL	B*15:10	[28]
TW10	p24 (108–117)	TSTLQEQIAW	B*57:01, B*57:03, B*58:01	[36]
KK9	p17 (18–26)	KIRLRPGGK	A*03:01	[19]
RK9	p17 (19–29)	RLRPGGKKK	A*03:01	
KW9	p17 (28–36)	KYRLKHLVW	A*24:02	
WF9	p17 (36–44)	WASRELEKF	B*35:01	
RY11	p17 (76–86)	RSLYNTVATLY	A*30:02	
KF11	p24 (30–40)	KAFSPEVIPMF	B*57:01, B*57:03, B*58:01	
YL9	p24 (164–172)	YVDRFFKTL	B*15:03, B*15:10, B*44:02	
SL9	p17 (77–85)	SLYNTIATL	A*02:01, A*02:02	
LF11	p17 (18–28)	LVWASRELERF	A*30, B*57	[37]
HL9	p24 (12–20)	HLVWASREL	C*08:04	
RY11	p17 (76–86)	RSLYNTVATLY	A*30	
LY9	p17 (78–86)	LFNTVATLY	A*29, B*44	
TL9	p24 (48–56)	TPQDLNTML	B*07, B*42:01, B*81:01	
DA9	p24 (166–174)	DRFYKTLRA	B*14	
YL9	p24 (164–172)	YVDRFFKTL	B*15	
WF9	p17 (36–44)	WASRELERF	B*35:01	
PF10	p24 (160–169)	PFRDYVDRFF	ND	
TD10	p24 (171–180)	TLRAEQATQD	C*03:04	
KF11	p24 (30–40)	KAFSPEVIPMF	B*57:01, B*57:03, B*58:01	[38]
ISW9	p24 (15–23)	ISPRTLNAW	B*57:01, B*57:03, B*58:01	
VF9	p24 (24–33)	VKVIEEKAF	B*15:03	[39]
ISW9	p24 (15–23)	ISPRTLNAW	B*57:03	[40]
TW10	p24 (108–117)	TSTLQEQIAW	B*57:03	
KF11	p24 (30–40)	KAFSPEVIPMF	B*57:03	
TW10	p24 (108–117)	TSTLQEQIAW	B*57:03, B*58:01	[41]
TL9	p24 (48–56)	TPQDLNTML	B*81:01	
KF11	p24 (30–40)	KAFSPEVIPMF	B*57:03	
LF11	p17 (18–28)	LVWASRELERF	B*57	
HA9	p24 (84–92)	HPVHAGPVA	B*35	
GI11	p24 (94–104)	GQMREPRGSDI	B*13	
LY9	p17 (78–86)	LYNTVATLY	A*29	
QW9	p24 (176–184)	QATQDVKNW	B*53:01	
NI9	p24 (193–201)	NANPDCKTI	B*08:01	
NL11	p24 (195–205)	NPDCKTILRAL	B*39:10	
RY11	p17 (76–86)	RSLYNTVATLY	A*30:02	
EL9	p17 (93–101)	EPKDREPL	B*08:01	
VF9	p24 (24–33)	VKVIEEKAF	B*15:03	
DI8	p24 (128–135)	DIYKRWII	B*08:01	
SL9	p17 (77–85)	SLYNTIATL	A*02:01	
GI11	p24 (94–104)	GQMREPRGSDI	B*13:02, C*06:02	[42]
RI9	p2p7p1p6 (66–74)	RQANFLGKI	B*13:02, C*06:02	
KF11	p24 (30–40)	KAFSPEVIPMF	B*57:03	[43]
TW10	p24 (108–117)	TSTLQEQIAW	B*58:01	
RM9	p17 (22–30)	RPGGKKHYM	ND	[19]
IL10	p17 (92–101)	IEVKDTKEAL	ND	
RY11	p17 (76–86)	RSLYNTVATLY	ND	
SL9	p17 (77–85)	SLYNTIATL	ND	
TL9	p24 (48–56)	TPQDLNTML	ND	
KF11	p24 (30–40)	KAFSPEVIPMF	B*57:01, B*57:03	[44]
TW10	p24 (108–117)	TSTLQEQIAW	B*57, B*58:01	[45]
ISW9	p24 (15–23)	ISPRTLNAW	B*57, B*58:01	
KF11	p24 (30–40)	KAFSPEVIPMF	B*57, B*58:01	
RY11	p17 (76–86)	RSLYNTVATLY	A*29:02, A*30:02	
TL9	p24 (48–56)	TPQDLNTML	B*81:01	
VF9	p24 (24–33)	VKVIEEKAF	B*15:03	
YL9	p24 (164–172)	YVDRFFKTL	B*15:10	
EL9	p24 (35–43)	EVIPMFTAL	A*26:01	
ISW9	p24 (15–23)	ISPRTLNAW	B*57:02, B*57:03	[46]
KF11	p24 (30–40)	KAFSPEVIPMF	B*57:03	
TW10	p24 (108–117)	TSTLQEQIAW	B*57:02, B*57:03, B*58:01	
TL9	p24 (48–56)	TPQDLNTML	B*81:01	
NY10	p24 (121–130)	NPPIPVGDIY	B*35:01	
DA9	p24 (166–174)	DRFYKTLRA	B*14:01	
QW9	p24 (176–184)	QASQEVKNW	B*58:01	
GL9	p24 (223–231)	GPSHKARVL	B*07:02	
TL9	p24 (48–56)	TPQDLNTML	B*81:01	[47]
TW10	p24 (108–117)	TSTLQEQIAW	B*57, B*58:01	
ISW9	p24 (15–23)	ISPRTLNAW	B*57:03	[48]
TW10	p24 (108–117)	TSTLQEQIAW	B*57:03	
KF11	p24 (30–40)	KAFSPEVIPMF	B*57:03	
FF8	ND	FPHFQQPF	B*08	[49]
NL9	ND	NVAPGPNAL	C*08:01	
CK9	ND	CLQPSDVSK	A*30:01	
SY9	ND	SSHANVKRY	A*01	
YL9	p24 (164–172)	YVDRFFKRL	C*03:03, C*03:04	[50]
TW10	p24 (108–117)	TSTLQEQIAW	B*57, B*58:01	[51]
ISW9	p24 (15–23)	ISPRTLNAW	B*57, B*58:01	
QW9	p24 (176–184)	QASQEVKNW	B*57, B*58:01	
KF11	p24 (30–40)	KAFSPEVIPMF	B*57, B*58:01	
AS9	p15 (1–10)	AEAMSQANS	B*45:01	[52]
TL9	p24 (48–56)	TPQDLNTML	B*42:01, B*81:01	
EL9	p17 (93–101)	EVIPMFTAL	A*26:38	
TW10	p24 (108–117)	TSTLQEQIAW	B*58:01	
YL9	p24 (164–172)	YVDRFFKTL	B*15:10	
ISW9	p24 (15–23)	ISPRTLNAW	B*57, B*58:01	[53]
KF11	p24 (30–40)	KAFSPEVIPMF	B*57, B*58:01	
QW9	p24 (176–184)	QASQEVKNW	B*57, B*58:01	
TW10	p24 (108–117)	TSTLQEQIAW	B*57, B*58:01	
TL9	p24 (48–56)	TPQDLNTML	B*81	[54]
QW9	p24 (176–184)	QASQEVKNW	B*57	
TW10	p24 (108–117)	TSTLQEQIAW	B*57, B*58:01	[55]
KF11	p24 (30–40)	KAFSPEVIPMF	B*57, B*58:01	
RY10	p17 (20–29)	RLRPGGKKKY	B*42:01	
SL9	p17 (77–85)	SLYNTIATL	A*30:02	
DL9	p24	DCKTILRAL	B*08:01	
TW10	p24 (108–117)	TSTLQEQIAW	B*57:02, B*57:03, B*58:01	[56]
TL9	p24 (48–56)	TPQDLNTML	B*42:01, B*81:01	
ISW9	p24 (15–23)	ISPRTLNAW	B*15:10, B*57:02, B*57:03	
GR11	p24 (8–18)	GQMVHQAISPR	A*74:01	[57]
QR10	p24 (9–18)	QMVHQAISPR	A*74:01	
KR9	p17 (12–20)	KLDKWEKIR	A*74:01	
KK10	p24 (131–140)	KRWIILGLNK	B*27:05	[24]
KF11	p24 (30–40)	KAFSPEVIPMF	B*57	
ISW9	p24 (15–23)	ISPRTLNAW	B*57	
SL9	p17 (77–85)	SLYNTIATL	A*02	
TL9	p24 (48–56)	TPQDLNTML	B*42:01, B*81:01	
DI8	p24 (128–135)	DIYKRWII	B*08:01	
LY9	p17 (78–86)	LFNTVATLY	A*29:02, B*44:03	
VF9	p24 (24–33)	VKVIEEKAF	B*15:03	
RY11	p17 (76–86)	RSLYNTVATLY	A*29, A*30	[58]
VF9	p24 (24–32)	VKVIEEKAF	B*15:03	
RY10	p17 (20–29)	RLRPGGKKHY	A*29	
ND	p24 (35–43)	EVIPMFTAL	A*26	
RL11	p24 (162–172)	RDYVDRFFKTL	B*44	
ND	p24 (44–52)	SEGATPQDL	B*44	
ND	p24 (175–184)	EQATQDVKNW	B*44	
ND	p24 (61–69)	GHQAAMQML	B*15:10	
DI8	p24 (128–135)	DIYKRWII	B*08	[15]
TL9	p24 (48–56)	TPQDLNTML	B*42, B*81, C*08	
QW9	p24 (176–184)	QASQEVKNW	B*57	
RY11	p17 (76–86)	RSLYNTVATLY	A*29, A*30	
VF9	p24 (24–33)	VKVIEEKAF	B*15:03	
LY9	p17 (78–86)	LFNTVATLY	A*29	
TW10	p24 (108–117)	TSTLQEQIAW	B*57	
KF11	p24 (30–40)	KAFSPEVIPMF	B*57	
AW11	p24 (174–184)	AEQASQEVKNW	B*44	
EL8	p2p7p1p6 (114–123)	EPKDREPL	B*08	
YL9	p24 (164–172)	YVDRFFKTL	C*03	
VL10	p24 (11–20)	VHQAISPRTL	B*15:10	
AF8	ND	ND	A*30	
KK9	p17 (18–26)	KIRLRPGGK	A*03:01	[22]
KW9	p17 (28–36)	KYRLKHIVW	A*24:02	
RY11	p17 (76–86)	RSLYNTVATLY	A*30:02, B*58, B*63	
LY9	p17 (78–86)	LFNTVATLY	A*29:02, B*44:03	
TK8	p17 (84–91)	TLYCVHQK	A*11:01	
IL10	p17 (92–101)	IEVKDTKEAL	B*40:01	
VL10	p24 (11–20)	VHQAISPRTL	B*15:10	
ISW9	p24 (15–23)	ISPRTLNAW	B*57:01, B*63	
KF11	p24 (30–40)	KAFSPEVIPMF	B*57:03	
EL9	p24 (35–43)	EVIPMFSAL	A*26:01	
TL9	p24 (48–56)	TPQDLNTML	B*07:02, B*39:10, B*42:01, B*81:01, C*08:02	
TW10	p24 (108–117)	TSTLQEQIAW	B*57:01, B*58:01	
NY10	p24 (121–130)	NPPIPVGDIY	B*35:01	
QW9	p24 (176–184)	QASQEVKNW	B*53:01, B*57:01	
RM9	p17 (22–30)	RPGGKKHYM	B*42:01, B*42:02	[14]
TL9	p24 (48–56)	TPQDLNTML	B*42:01	
GL9	p24 (223–231)	GPSHKARVL	B*42:01	
GF9	p2p7p1p6 (22–30)	GPKRIVKCF	B*42:01	
ISW9	p24 (15–23)	ISPRTLNAW	B*57:02, B*57:03	[13]
KF11	p24 (30–40)	KAFSPEVIPMF	B*57:03	
TW10	p24 (108–117)	TSTLQEQIAW	B*57:02, B*57:03, B*58:01	
QW9	p24 (176–184)	QASQEVKNW	B*57:02, B*57:03, B*58:01	
LF11	p17 (18–28)	LVWASRELERF	B*57:03	
DW10	p24 (71–80)	DTINEEAAEW	B*58:01	
HA9	p24 (84–92)	HPVHAGPVA	B*35:01	[59]
NY10	p24 (121–130)	NPPIPVGDIY	B*35:01	
PY9	p24 (122–130)	PPIPVGEIY	B*35:01	
TL9	p24 (48–56)	TPQDLNTML	B*39:10, B*81:01	[24]
TW10	p24 (108–117)	TSTLQEQIAW	B*58:01	[60]
TA10	p24 (107–116)	TTSTLQEQIA	A*68:02	[61]
TL9	p24 (48–56)	TPQDLNTML	B*81:01	[62]
ISW9	p24 (15–23)	ISPRTLNAW	B*57:03	
YL9	p24 (164–172)	YVDRFFKTL	B*15:10	
TW10	p24 (108–117)	TSTLQEQIAW	B*57:03, B*58:01	
GL9	p24 (223–231)	GPSHKARVL	B*07:02	[63]
SV9	p24 (16–24)	SPRTLNAWV	B*07:02	
RM9	p17 (22–30)	RPGGKKHYM	B*07:02	
TL9	p24 (48–56)	TPQDLNTML	B*07:02	
HA9	p24 (84–92)	HPVHAGPVA	B*07:02	
KF11	p24 (30–40)	KAFSPEVIPMF	B*57:03	[64]
HA9	p24 (84–92)	HPVHAGPVA	B*42	[65]
DW10	p24 (71–80)	DTINEEAAEW	B*57	
ISW9	p24 (15–23)	ISPRTLNAW	B*57, B*58	
RY11	p17 (76–86)	RSLYNTVATLY	A*30:02	
LY9	p17 (78–86)	LFNTVATLY	A*29	
SL9	p17 (77–85)	SLYNTIATL	A*02	
TL9	p24 (48–56)	TPQDLNTML	B*07:02, B*42:01, B*81:01	[66]
TW10	p24 (108–117)	TSTLQEQIAW	B*57	[67]
TL9	p24 (48–56)	TPQDLNTML	B*42	
EV9	p17 (74–82)	ELRSLYNTV	B*08	
VF9	p24 (24–33)	VKVIEEKAF	B*15:03	
YL9	p24 (164–172)	YVDRFFKRL	C*03	
HW9	p17	HYMLKHLVW	A*23	
RY11	p17 (76–86)	RSLYNTVATLY	A*30:02	
AW11	p24 (174–184)	AEQASQEVKNW	B*44	
RY10	p17 (20–29)	RLRPGGKKHY	A*03	
SL9	p17 (77–85)	SLYNTIATL	A*02	
TW10	p24 (108–117)	TSTLQEQIAW	B*57, B*58:01	[68]
QW9	p24 (176–184)	QASQEVKNW	B*57, B*58:01	
NL11	p24 (195–205)	NPDCKTILRAL	B*39:10, B*58:01	
EL8	p2p7p1p6 (114–123)	EPKDREPL	B*08	
ISW9	p24 (15–23)	ISPRTLNAW	B*57	
WF9	p17 (36–44)	WASRELERF	B*57	
EI8	p24 (128–135)	EIYKRWII	B*08	
KF11	p24 (30–40)	KAFSPEVIPMF	B*57	
GL9	p24 (223–231)	GPSHKARVL	B*07, B*39:10	
TL9	p24 (48–56)	TPQDLNTML	B*39:10, B*81:01	
DI8	p24 (128–135)	DIYKRWII	B*08	
SL9	p17 (77–85)	SLYNTIATL	B*44	
SK10	p17 (6–15)	SILRGGKLDK	A*74	[69]
TL9	p24 (48–56)	TPQDLNTML	B*42:01, B*81:01	
YL9	p24 (164–172)	YVDRFFKRL	C*03	
DA9	p24 (166–174)	DRFYKTLRA	B*14:02	[70]
TL9	p24 (48–56)	TPQDLNTML	B*42:01, B*81:01	[71]
SL9	p17 (77–85)	SLYNTIATL	ND	[72]

Abbreviations: CTL, Cytotoxic T‐lymphocyte; et al., and others; Gag, Group‐specific antigen; HIV‐1, Human Immunodeficiency Virus type 1; HLA‐I, Human Leukocyte Antigen class I; ND, not documented.

In the capsid protein (p24), TPQDLNTML (TL9, Gag p24 48–56) was more prevalent throughout the studies at 16.00%, followed by TSTLQEQIGW (TW10, Gag p24 108–117) at 13.3%, KAFSPEVIPMF (KF11, Gag p24 30–40) at 12.00%, ISPRTLNAW (ISW9, Gag p24 15–23) at 9.33%, YVDRFFKTL (YL9, Gag p24 164–172) at 8.00%, and QASQEVKNW (QW9, Gag p24 176–184) at 6.00%. The remainder of the epitopes were detected at frequencies less than 5% (Table 2, Figure 3).

There were a lower number of epitopes detected for the Gag polyprotein (p2p7p1p6) when compared to the matrix and capsid proteins. However, within p2p7p1p6, EPKDREPL (EL8, Gag p2p7p1p6 114–123) was more frequent (40.00%), while the remaining epitopes, RQANFLGKI (RI9, Gag p2p7p1p6 66–74), GPKRIVKCF (GF9, Gag p2p7p1p6 22–30), and AEAMSQANS (AS9, Gag p15) appeared at equal proportions of 20.00% (Table 2, Figure 3).

Interestingly, most HIV‐1 subtype C epitopes from the southern African studies are not listed on the LANL HIV database and this includes epitopes such as PFRDYVDRFF (PF10, Gag p24 160–169), TLRAEQATQD (TD10, Gag p24 171–180), FPHFQQPF (FF8, Gag), NVAPGPNAL (NL9, Gag), CLQPSDVSK (CK9, Gag), SSHANVKRY (SY9, Gag), AEAMSQANS (AS9, Gag p15 1–10), DCKTILRAL (DL9, Gag p24), EVIPMFTAL (Gag p24 35–43), SEGATPQDL (Gag p24 44–52), EQATQDVKNW (Gag p24 175–184), GHQAAMQML (Gag p24 61–69), VHQAISPRTL (VL10, Gag p24 11–20), Gag AF8, and HYMLKHLVW (HW9, Gag p17) which were previously described for subtype C cohorts [13, 15, 22, 33, 34, 35, 36, 37, 38].

### 3.4 HLA‐I Typing and Functional Assays

The majority of studies (85.71%) performed HLA‐I typing. The most frequently used functional assay to detect CTL epitopes was IFN‐γ ELISpot (65.15%) followed by tetramer staining (16.67%), intracellular cytokine staining (ICS) (10.60%), and Chromium release assay (7.58%) (Table 1).

**FIGURE 4:**
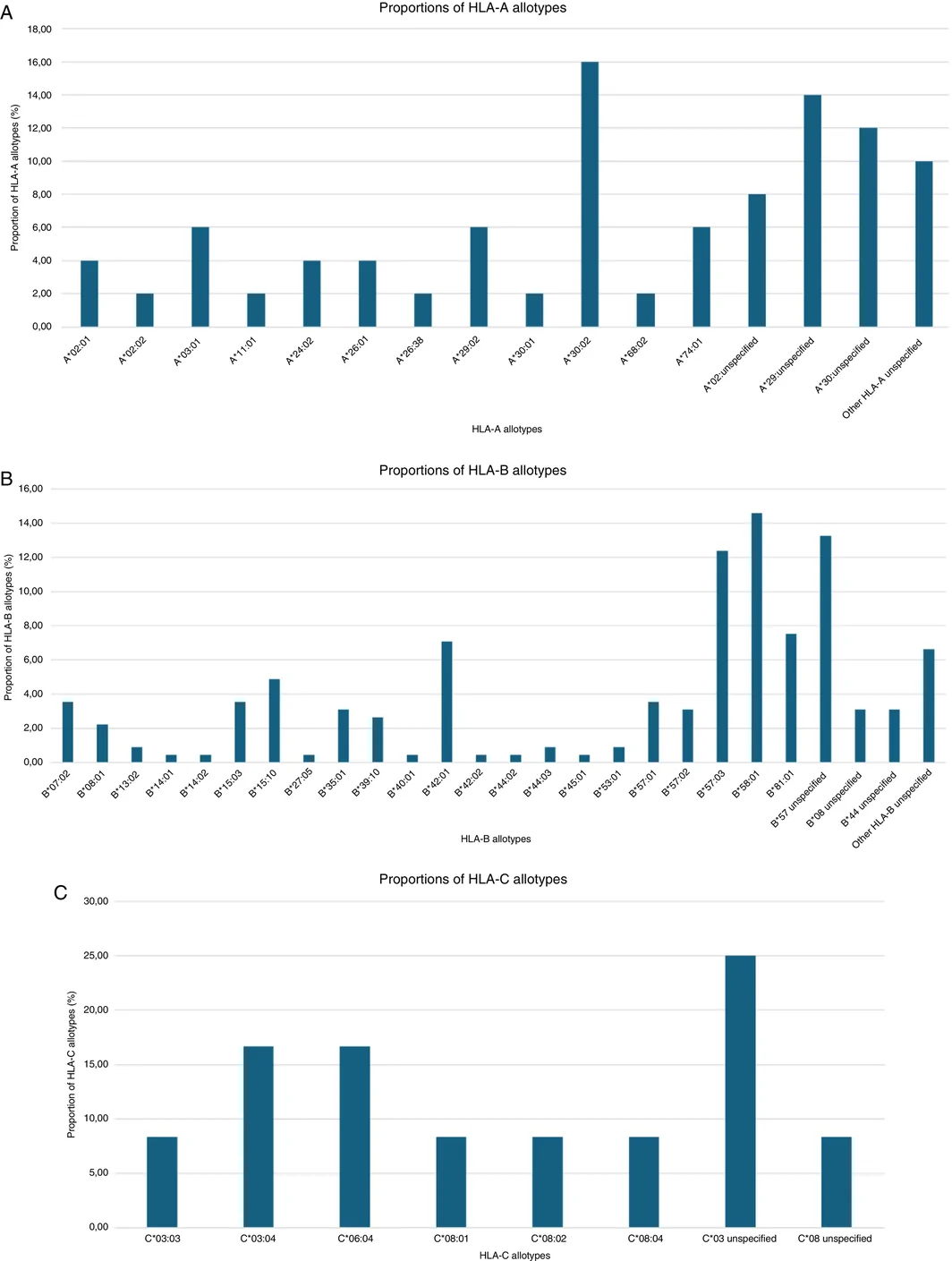
Proportion of HLA‐I allotypes from studies in the southern African region; (A) HLA‐A allotypes, (B) HLA‐B allotypes, and (C) HLA‐C allotypes. HLA, Human Leukocyte Antigen.

The reporting of HLA‐A, ‐B and ‐C allotypes in this systematic review was 18%, 78%, 4%, respectively. Among HLA‐A allotypes, HLA‐A*30:02 was the most commonly detected at 16.00%, followed by HLA‐A*03:01, −A*29:02, and ‐A*74:01 at equal proportions of 6.00% each. The most frequently detected HLA‐B allotypes were HLA‐B*58:01 (14.60%), followed by ‐B*57:03 (12.39%), ‐B*81:01 (7.52%), and ‐B*42:01 (7.08%). HLA‐C*03:04 and ‐C*06:02 were the most frequently detected HLA‐C allotypes and occurred at equal proportions of 16.67%, followed by ‐C*03:03, ‐C*08:01, ‐C*08:02, and ‐C*08:04 at equal proportions of 8.33%. Some studies did not provide high‐resolution data on HLA‐I allotypes but only reported on HLA‐I serotypes. The commonly reported HLA‐I serotypes were HLA‐A*29, −A*30, ‐B*57, ‐C*03 and ‐C*08 (Table 2, Figure 4).

The common HLA‐B allotypes; HLA‐B*57, ‐B*58:01, ‐B*42:01 and ‐B*81:01 restricted the following Gag CTL epitopes: TSTLQEQIGW (TW10, Gag p24 108–117), ISPRTLNAW (ISW9, Gag p24 15–23), KAFSPEVIPMF (KF11, Gag p24 30–40), and TPQDLNTML (TL9, Gag p24 48–56), respectively. HLA‐A*30:02, −A*29 and ‐A*02 allotypes restricted the following Gag CTL epitopes: RSLYNTVATLY (RY11, Gag p17 76–86), LFNTVATLY (LY9, Gag p17 78–86) and SLYNTIATL (SL9, Gag p17 77–85), respectively. HLA‐C*03, ‐C*06:02, and ‐C*08 allotypes restricted YVDRFFKTL (YL9, Gag p24 164–172), RQANFLGKI (RI9, Gag p2p7p1p6 66–74), and HLVWASREL (HL9, Gag p24 12–20) epitopes, respectively (Table 2, Figure 4).

## 4 Discussion

To our knowledge, this is the first systematic review to report on HIV‐1 subtype C Gag CTL epitopes in the southern African region. This review shows that the majority of studies (58.44%) were conducted in South Africa. Possible reasons for this could include factors such as availability of funding for research and increased collaboration with institutions in high income countries [39, 40, 41].

During early HIV‐1 infection, robust CTL responses are mostly directed to immunodominant Gag p24 epitopes to reduce HIV‐1 viraemia to a VL set‐point. Studies have shown that within the Gag protein, p24 was the most frequently targeted region, and increased magnitude and breadth of CTL response directed to p24 epitopes coincided with reduction in HIV‐1 viraemia. Moreover, the breadth and magnitude of CTL responses targeting p24 epitopes were higher compared to those targeting p17 as well as p2p7p1p6 epitopes [19, 34, 38, 42, 43, 44, 45]. This review exposed that some CTL epitopes identified in the southern African studies are not reported on the LANL HIV database. This highlights a need to have the LANL HIV database updated with this information as this database is used as a reference for CTL epitopes [13, 15, 22, 33, 34, 35, 36, 37, 38].

IFN‐γ ELISpot (Interferon‐gamma enzyme‐linked immunospot assay) which quantifies the secretion of cytokines such as IFN‐γ by CTLs following stimulation with a specific antigen, was the most frequently used functional assay. IFN‐γ ELISpot is considered the gold standard for the characterisation of HIV‐1 specific CTL epitope responses, as it is known to be more sensitive, while allowing for the screening of a large cohort of participants, when compared to other flow cytometry‐based functional assays such as tetramer staining and intracellular cytokine staining (ICS), as well as Chromium‐51 release assay which are time‐consuming and have complex workflows requiring specialised staff [42, 46, 47, 48, 49, 50].

*HLA‐B* alleles are associated with better control of HIV‐1 infection compared to *HLA‐A* and *HLA‐C* alleles [14, 51]. Kiepiela et al., found that a significantly higher number of CTL responses were restricted by HLA‐B allotypes compared to HLA‐A allotypes. Moreover, they found that HLA‐B allotypes imposed more selection pressure on HIV‐1 compared to HLA‐A allotypes and were associated with either higher or lower viral loads and influenced CD4+ T cell counts, depending on whether they were protective or disease susceptible [28]. *HLA‐B* alleles evolve more rapidly and show greater allele diversity compared to *HLA‐A* and *HLA‐C* alleles. This genetic diversity within *HLA‐B* alleles is influenced by intra‐locus recombination events within exon 2 and 3, which encodes the peptide binding region. These recombination events can reshuffle the amino acid (aa) residues which make up the C, D and F binding pockets, thus affecting the peptide repertoire presented by HLA‐B molecules [14, 15, 28, 51, 52, 53, 54]. This review observed that in the southern African region, HLA‐B*57 and ‐B*58:01 were more commonly associated with control of HIV‐1 [16, 28, 53, 55]. Interestingly, Tshabalala et al. observed a high prevalence of HLA‐B*57:03 and ‐B*58:01 among black populations in the southern African region, and this is more consistent with the findings of this review [29].

During early HIV‐1 infection, immunodominant TL9 has been shown to be hierarchically targeted by HLA‐B*81:01, B*39:10, and B*42:01 allotypes, whereas TW10 and ISW9 are both targeted by HLA‐B*57, and ‐B*58:01 allotypes, to reduce HIV‐1 viraemia to a low set‐point [28, 42, 55, 56]. Kløverpris et al. found that a minority of HLA‐B*42:01‐positive participants also targeted the immunodominant GF9 epitope derived from Gag p2p7p1p6, and that responses directed to this epitope coincided with reduced HIV‐1 viraemia [14]. The emergence of immune escape variants in immunodominant Gag p24 epitopes is often associated with replicative fitness costs to the HIV‐1 virus which further contributes to the control of viraemia. However, compensatory mutations are known to partially or fully restore replication fitness. Following antigen presentation by HLA‐I molecules, CTLs are stimulated to secrete proinflammatory cytokines such as interferon gamma (IFN‐γ) and granules which contain granzymes and perforin. These signalling molecules can trigger cellular processes such as apoptosis, which contribute to the antiviral response, limiting viral replication and thus controlling the spread of the infection [24, 38, 57, 58, 59]. In chronic HIV‐1 infection, the hierarchy shifts to p24 KF11 which is restricted by HLA‐B*57:03, and to p17 epitopes such SL9 and RY11 which are restricted by HLA‐A*02 and ‐A*30:02, respectively [19, 48, 56, 60]. Additionally, Honeyborne et al. showed that immunodominant Gag p2p7p1p6 epitope, RI9 which is restricted by HLA‐B*13 allotypes is mainly targeted during chronic infection [61].

HLA‐I micro‐polymorphism refers to minor or fewer aa variation(s) within similar HLA‐I allotypes, which can affect HLA‐epitope binding preferences. For instance, HLA‐B*42:01 and ‐B*42:02 alleles from the *B7* supertype family differ by a single aa at the α1 helix peptide binding groove, and this leads to differences in CTL epitope recognition and control of HIV‐1 [14, 24, 62]. Individuals carrying HLA‐B*42:01 have been shown to restrict immunodominant Gag p24‐TL9, leading to lower viral load set points compared to those who carry HLA‐B*42:02. Additionally, HLA‐B*42:02‐positive participants do not target Gag p24 epitopes, which would explain the inability to achieve low viral load set‐points, as mutations within Gag p24 have been shown to cripple viral replication, leading to better control of HIV‐1 viraemia [14, 16, 27, 63].

HLA‐B*58:01 varies from ‐B*58:02 by three amino acids and as a result, ‐B*58:02 has reduced space in the F pocket compared to HLA‐B*58:01 and is thus unable to bind large hydrophobic aa residues. This may explain why HLA‐B*58:02 has a reduced efficiency in the presentation of peptide epitopes to CTLs, leading to poor control of HIV‐1 viraemia and faster disease progression. Contrary to HLA‐B*58:02, HLA‐B*58:01 allotype, which is more similar to HLA‐B*57:01, has been observed to restrict immunodominant ISW9 and TW10 and thus leading to slower disease progression [36, 48]. Similar to HLA‐I micro‐polymorphism observed with HLA B42 and B58 serotypes, HLA‐A*30:01 and ‐A*30:02 vary at the carboxyl‐terminal peptide anchor residue leading to differences in peptide binding characteristics and peptide presentation to CTLs, and disease outcomes [48, 58, 65].

The immune pressure exerted by Gag CTL responses leads to selection of viral escape mutations. *HLA‐B7* supertype alleles such as HLA‐B*39:10, ‐B*42:01, and ‐B*81:01 are known to be protective against HIV‐1 subtype C and have been shown to restrict Gag p24‐TL9 [33, 42, 48, 66, 67]. The degree of immune selection pressure varies between the three allotypes with HLA‐B*81:01 being more dominant than HLA‐B*39:10, and lastly HLA‐B*42:01 [24, 25]. In vitro, HLA‐B*81:01 has been shown to select for T186S mutation, which is strongly associated with reduced viral replication capacity, and in turn may explain the low viral load that is observed in HLA‐B*81:01‐positive participants with subtype C viral infection [24, 65, 68]. HLA‐B*81:01‐associated compensatory mutations, Q182S and T190X, were shown to partially restore replication capacity [65, 68, 69].

Immune selection pressure mediated by HLA‐B*57/B*58:01 selects for the T242N mutation in TW10 and the A163X (X = D, S, G or N) mutation in KF11, which decreases replicative fitness of the virus and that associates with lower viral loads, and improved CD4^+^ T cell counts in subtype C viral infection [36]. Furthermore, the T242N mutation has been observed to persist in HLA‐B*57/B*58:01‐positive individuals for a period of at least 8 years. The compensatory mutation H219X (X = Q, P or R) can occur along with T242N but can also be present in the absence of T242N, suggesting that this compensatory mutation does not fully restore replication capacity of the virus. In chronically infected HLA‐B*57/B*58:01‐negative individuals, T242N is rare and is known to rapidly revert to wild‐type within 6–24 months following transmission from HLA‐B*57/B*58:01‐positive donors [36, 55]. Interestingly, in the absence of HLA‐B*57/B*58:01, no reversions were observed for A163X in the KF11 epitope. CTL responses to the ISW9 epitope select for A146X (X = *P* or S), which does not affect replication fitness of the virus, and in addition, following transmission to HLA‐B*57/B*58:01‐negative individuals, and reversions to wild‐type have only been observed in a minority of study participants [36, 52, 55, 66, 70].

The role of *HLA‐C* alleles in HIV‐1 disease progression remains unclear, as in most studies, which have assessed the magnitude and breadth of CTL responses against HIV‐1 Gag, *HLA‐C* alleles are invariably reported to be in close linkage disequilibrium (LD) with *HLA‐B* alleles [16, 28, 33, 71]. LD refers to the non‐random association of *HLA‐I* alleles at two or more gene loci, and this is known to influence the magnitude and breadth of CTL responses targeted at Gag epitopes. In southern African populations, HLA‐B*57 and ‐B*58:01 have been observed to be in strong LD with HLA‐C*07 alleles, however, in these cohorts, there were no significant differences observed in the polyfunctionality of CTL responses. In addition, HLA‐B*58:02, and ‐B*81:01 have been observed to be in strong LD with HLA‐C*06:02, and HLA‐C*04:01, respectively [27, 28, 51]. Interestingly, in a subtype C infection cohort, it was observed that study participants carrying HLA‐B*42:01 in strong LD with HLA‐A*30:01 and ‐C*17:01 were able achieve a significantly lower HIV‐1 viral load compared to carriers of HLA‐B*42:02 also in strong LD with HLA‐A*30:01 and ‐C*17:01 [14, 48].

CD8^+^ T cell immune responses targeted at immunodominant epitopes located within *gag* have been shown to be associated with control of HIV viraemia, and other studies highlight that *gag* epitopes are the primary target in elite controllers [72, 73]. This highlights the importance of making more efforts to understand immunodominant HIV‐1 Gag CTL epitopes targeted during natural HIV‐1 infection as this can potentially contribute to the development of HIV vaccines [73]. Most prophylactic HIV‐1 vaccine candidates tested in clinical trials have included Gag epitopes in their design in an attempt to elicit strong CTL responses [74, 75, 76, 77, 78, 79, 80, 81, 82, 83, 84]. This systematic review sheds some insights on commonly recognised Gag CTL epitopes in the southern African region, which could be considered for inclusion in a vaccine construct for better representation of subtype C in an HIV‐1 vaccine. This would ensure that an effective HIV‐1 vaccine works optimally for prevention of subtype C infections, as this subtype constitutes ~50% of the global infections [85, 86, 87].

Data from this systematic review might also contribute to the design of HIV‐1 therapeutic vaccines, which are being developed for use in PLWH for controlling HIV‐1 without the need of using ART [88, 89, 90, 91]. These vaccines can be used on their own for achieving functional cure or in combination with other drugs as part of an HIV‐1 cure strategy [91, 92]. Gag CTL epitopes have been included in some candidate HIV‐1 therapeutic vaccines to elicit strong CTL responses. These have shown good efficacy in the reduction of HIV‐1 reservoirs following analytical treatment interruption when used as part of an HIV‐1 cure strategy [90, 91, 92, 93, 94].

Limitations of this review are that the search was limited to only peer‐reviewed publications from three databases, which could have limited the search results; and that most studies were conducted in South Africa. Lack of or insufficient data from other southern African countries may have influenced the findings of this systematic review. HLA‐I typing was not performed in some studies, in addition, some studies only referred to HLA‐I serotypes and therefore high‐resolution HLA‐I allele typing was not available for comparisons; these may have limited the evaluation of the frequency of protective versus disease‐susceptible HLA‐I allotypes. In addition, participants' race and sex were also not reported in most studies.

## 5 Conclusion and Future Directions

This systematic review important insights into the frequencies of immunodominant Gag epitopes and HLA class I allotypes that influence the control of HIV‐1 viraemia in the southern African region. In this region, protective HLA‐B*57, and ‐B*58:01 allotypes, which restrict TW10, and ISW9 epitopes, and HLA‐B*42:01 and ‐B*81:01 allotypes which restrict the TL9 epitope, have been shown to be more commonly associated with better control of HIV‐1 viraemia, whereas disease susceptible allotypes including HLA‐B*42:02 and ‐B*58:02 were associated with faster progression to AIDS. This review also observed that most HIV‐1 subtype C epitopes described in southern African studies are not listed on LANL HIV database and highlights a need to have this database updated with this information. This systematic review could provide insights into the design of an HIV‐1 vaccine that could be more effective in the southern African region when immunodominant epitopes and the corresponding restricting HLA‐I allotypes are taken into account. We propose that future research should consider the following; (1) expanding research to areas of the southern Africa region where there is lack of HLA and Gag CTL epitope data, (2) evaluating recognition of Gag CTL epitopes in patients on ART in order to identify potential targets of therapeutic vaccines that may be considered for use as part of HIV‐1 cure strategies, (3) comprehensive evaluation of immune responses including both innate and adaptive responses, and (4) prioritising the sub‐Saharan African region for both HIV‐1 vaccine and cure research due to high burden of HIV‐1 infections in this region.

## Author Contributions

Conceptualization: Simnikiwe H. Mayaphi. Methodology: Tumelo L. Fortuin, Simnikiwe H. Mayaphi. Funding acquisition: Tumelo L. Fortuin, Caroline T. Tiemessen, Simnikiwe H. Mayaphi. Investigation: Tumelo L. Fortuin, Shayne Loubser, Paballo Nkone, Simnikiwe H. Mayaphi. Supervision: Paballo Nkone, Caroline T. Tiemessen, Simnikiwe H. Mayaphi. Project administration: Tumelo L. Fortuin, Simnikiwe H. Mayaphi. Data analysis: Tumelo L. Fortuin, Paballo Nkone, Shayne Loubser, Caroline T. Tiemessen, Simnikiwe H. Mayaphi. Writing – original draft: Tumelo L. Fortuin. Writing – review and editing: Tumelo L. Fortuin, Paballo Nkone, Shayne Loubser, Caroline T. Tiemessen, Simnikiwe H. Mayaphi.

## Funding

TL was funded by the National Research Foundation (NRF) and Poliomyelitis Research Foundation (# 24/30). SHM received funding from Poliomyelitis Research Foundation (#23/69), National Research Foundation, and University of Pretoria Research Development Fund. CTT receives funding through the South African Research Chairs Initiative of the Department of Science and Innovation and National Research Foundation of South Africa (84177).

## Ethics Statement

Approval (reference number—165/2024) was obtained for this study from the Faculty of Health Sciences Research Ethics Committee, University of Pretoria, South Africa.

## Conflicts of Interest

The authors declare no conflicts of interest.

## Data Availability

All data generated or analysed during this study are included in this manuscript.
